# Evaluating the Effectiveness of Neuroprotective Strategies in Enhancing Post-stroke Recovery: A Systematic Review of Meta-Analyses and Clinical Trials

**DOI:** 10.7759/cureus.71343

**Published:** 2024-10-12

**Authors:** Baran Dilshad Hassan, Muath M Dabas, Kimberly Kanemitsu, Nuzhat Faran, Tajammul Abbas

**Affiliations:** 1 College of Medicine, Hawler Medical University, Erbil, IRQ; 2 General Surgery, University of Jordan, Amman, JOR; 3 Clinical Sciences, Windsor University School of Medicine, Chicago, USA; 4 Internal Medicine, Fatima Memorial Hospital, Lahore, PAK; 5 Internal Medicine, Nishtar Medical University, Multan, PAK

**Keywords:** cerebrolysin, clinical trials, ischemic stroke, lithium, meta-analysis, neuroprotection, normobaric oxygen, ssris, stroke recovery, systematic review

## Abstract

This systematic review evaluates the effectiveness of various neuroprotective strategies in enhancing recovery following acute ischemic stroke, focusing on interventions such as normobaric oxygen (NBO), lithium, selective serotonin reuptake inhibitors (SSRIs), and Cerebrolysin. Drawing upon data from six primary studies, including randomized controlled trials (RCTs) and meta-analyses, we assessed these therapies' impact on functional outcomes, motor recovery, and neurological improvement. Normobaric oxygen, across 12 RCTs, demonstrated limited efficacy in improving recovery outcomes or reducing mortality. Lithium, supported by animal models but with inconclusive human data, showed potential in reducing stroke volume but did not significantly enhance functional recovery in clinical trials. SSRIs, particularly fluoxetine, showed moderate success in improving motor recovery, as evidenced by the FLAME (Fluoxetine for Motor Recovery after Acute Ischaemic Stroke) trial and meta-analyses. Cerebrolysin demonstrated consistent improvement in early neurological function and motor recovery, with a number-needed-to-treat (NNT) of 7.1 for early NIHSS (National Institutes of Health Stroke Scale) score improvements. Our Preferred Reporting Items for Systematic Reviews and Meta-Analyses (PRISMA)-guided search covered PubMed, Medline, Embase, and the Cochrane Library up to September 2024. These findings emphasize the mixed efficacy of these neuroprotective interventions and underscore the necessity for personalized treatment protocols and further large-scale, controlled trials to clarify their roles in clinical practice. This review contributes to the ongoing dialogue on optimizing post-stroke recovery and highlights the critical need for evidence-based neuroprotective strategies.

## Introduction and background

Stroke remains one of the leading causes of death and disability worldwide, with ischemic stroke accounting for the majority of cases. The pathophysiology of stroke involves a cascade of events, including excitotoxicity, oxidative stress, inflammation, and apoptosis, all contributing to brain injury and long-term neurological deficits [1]. Early intervention with neuroprotective therapies has the potential to limit such damage and enhance functional recovery [2]. Despite significant advancements in acute stroke care, such as thrombolysis and thrombectomy, there is still an unmet need for effective neuroprotective strategies that can be implemented during the subacute phase of stroke recovery [3].

We selected normobaric oxygen (NBO), lithium, selective serotonin reuptake inhibitors (SSRIs), and Cerebrolysin for review due to their unique mechanisms of action and their emerging roles in neuroprotection and post-stroke recovery. These therapies have shown varying degrees of promise in both preclinical studies and early-phase clinical trials. NBO has been explored for its ability to increase oxygen delivery to ischemic brain tissue, though meta-analyses suggest that it may not significantly improve functional outcomes [4,5]. Lithium, widely known for its mood-stabilizing effects, has demonstrated potential in reducing stroke volume and improving post-stroke function in animal models, but human data remain inconclusive. SSRIs, particularly fluoxetine, have garnered attention for their ability to enhance motor recovery post-stroke, independent of their antidepressant effects, as supported by the FLAME (Fluoxetine for Motor Recovery After Acute Ischemic Stroke) trial and subsequent meta-analyses [6]. Cerebrolysin, a neuropeptide preparation, has been the subject of multiple randomized controlled trials (RCTs) and meta-analyses, with studies suggesting its potential for early neurological improvement, though evidence of long-term benefits remains limited [7].

These four interventions were selected based on their clinical relevance, availability of substantial data from RCTs and meta-analyses, and their distinct neuroprotective mechanisms. Compared to other emerging interventions, such as hypothermia, neurotrophic factors, and stem cell therapies, NBO, lithium, SSRIs, and Cerebrolysin have garnered the most clinical attention and research, making them well-suited for a focused systematic review. The objective of this review is to evaluate the efficacy of these neuroprotective strategies in improving post-stroke recovery, particularly in terms of functional outcomes, motor recovery, and neurological improvement. By synthesizing data from clinical trials and meta-analyses, this review aims to provide a clearer understanding of the benefits and limitations of these therapies in stroke rehabilitation.

## Review

Materials and methods

Search Strategy

Our search strategy was developed following the Preferred Reporting Items for Systematic Reviews and Meta-Analysis (PRISMA) guidelines to examine the efficacy of emerging neuroprotective strategies in enhancing post-stroke recovery [8]. We performed comprehensive searches across multiple electronic databases including PubMed, Medline, Embase, and the Cochrane Library, spanning from the inception of each database up to September 2024. A combination of keywords and Medical Subject Headings (MeSH) terms was used to frame our search query, incorporating terms such as "acute ischemic stroke", "neuroprotection", "normobaric oxygen", "lithium", "selective serotonin reuptake inhibitors", "Cerebrolysin", and "clinical recovery". Boolean operators ('AND', 'OR') were utilized to refine the search and ensure an exhaustive retrieval of pertinent literature. Example search strings included: "acute ischemic stroke AND neuroprotective strategies AND clinical outcomes", and "normobaric oxygen OR lithium OR SSRIs OR Cerebrolysin AND stroke recovery".

To capture a wider array of studies and maximize the inclusion of relevant data, we also scrutinized the reference lists of all retrieved articles for potential sources that might have been missed in the initial database search. Additionally, we explored clinical trial registries and relevant academic conference proceedings to uncover any unpublished or ongoing studies that could provide insights into the effectiveness of these interventions. The search was limited to studies published in English and involved only those that were peer-reviewed. Our inclusion criteria targeted clinical trials, meta-analyses, and randomized controlled trials that specifically assessed the impact of neuroprotective interventions on motor recovery, functional outcomes, and neurological status in patients following an acute ischemic stroke. This meticulous approach ensured a robust and comprehensive search, underpinned by expert review to validate the relevance and quality of the selected sources.

Eligibility Criteria

The eligibility criteria for this systematic review are precisely defined to ensure the inclusion of rigorous and pertinent studies examining the effectiveness of neuroprotective strategies for enhancing recovery post-stroke. We focus on peer-reviewed research articles, including clinical trials, RCTs, and meta-analyses, that specifically evaluate neuroprotective interventions in patients following an acute ischemic stroke. The interventions of interest include normobaric oxygen therapy, lithium administration, SSRIs, and Cerebrolysin treatment. To be included, studies must present clear outcomes related to neurological improvement, functional recovery, or mortality rates. Our inclusion criteria mandate that the studies are published in English, peer-reviewed, and available from the inception of each database up to the current date to ensure comprehensive coverage of both historical and recent advancements in stroke recovery research.

Exclusion criteria are designed to refine the scope of our review and exclude studies that do not directly address the specific question of neuroprotective efficacy in stroke recovery. Studies that do not involve acute ischemic stroke patients, such as those focusing on hemorrhagic stroke or unrelated neurological conditions, are excluded. Research articles that do not evaluate the specified interventions (normobaric oxygen, lithium, SSRIs, Cerebrolysin) or do not have clear outcome measures related to stroke recovery are also omitted. Additionally, non-peer-reviewed articles, conference abstracts, editorials, and commentaries are excluded to maintain the scientific rigor of the review. Non-English studies are excluded due to potential translation inaccuracies that could affect the interpretation of data. This stringent selection process ensures that only the most relevant and high-quality studies are included in our systematic review, providing reliable insights into the efficacy of neuroprotective strategies in stroke rehabilitation.

Data Extraction

Our data extraction process was meticulously structured to capture and validate essential data for our systematic review of the efficacy of neuroprotective strategies in enhancing recovery following an acute ischemic stroke. The process began with an initial screening of articles based on titles and abstracts, conducted by two independent reviewers who assessed relevance and classified studies accordingly. Articles deemed potentially relevant underwent a detailed full-text review.

For consistency and accuracy, we employed a standardized data extraction form designed in Microsoft Excel (Microsoft Corp., Remond, WA). Reviewers independently recorded critical details such as the lead author’s name, publication year, study design, sample size, intervention details, primary outcomes, key findings, and study limitations. To manage discrepancies between reviewers, any disagreements were first discussed between the two reviewers. If consensus could not be reached, a third reviewer was consulted to mediate and provide a final decision. This method ensured that discrepancies were resolved transparently and consistently.

In terms of impact on the data synthesis process, discrepancies were minimal and typically involved differences in interpretation of study outcomes or study quality. These were resolved before the data synthesis phase, ensuring that all extracted data were complete and harmonized. As a result, the final dataset used for synthesis was comprehensive and reliable, with minimal impact from the initial discrepancies. This rigorous approach facilitated an accurate and thorough synthesis of the current evidence on neuroprotective interventions for stroke recovery.

Data Analysis and Synthesis

Given the variability in interventions and outcomes among the studies on neuroprotective strategies for stroke recovery, a meta-analysis was deemed inappropriate. Instead, our review adopted a qualitative synthesis approach to explore and integrate the findings effectively. We systematically categorized the key results from each study to identify recurring themes and notable distinctions concerning the neuroprotective effects, methodologies, and patient outcomes. This thematic synthesis allowed us to deeply examine the nuances of each intervention's effectiveness, such as normobaric oxygen, lithium, SSRIs, and Cerebrolysin, across different clinical settings and stroke severities. Our narrative synthesis consolidated these insights, providing a comprehensive overview of the current landscape of neuroprotective strategies in stroke rehabilitation. We discussed the implications of these findings within the wider context of stroke recovery management, pinpointed existing gaps in research, and proposed directions for future investigations. This methodological approach not only underscored the diverse effects of these therapies but also highlighted the quality and robustness of the existing evidence, thus contributing valuable perspectives to the field of stroke recovery research.

Results

Study Selection Process

**Figure 1:**
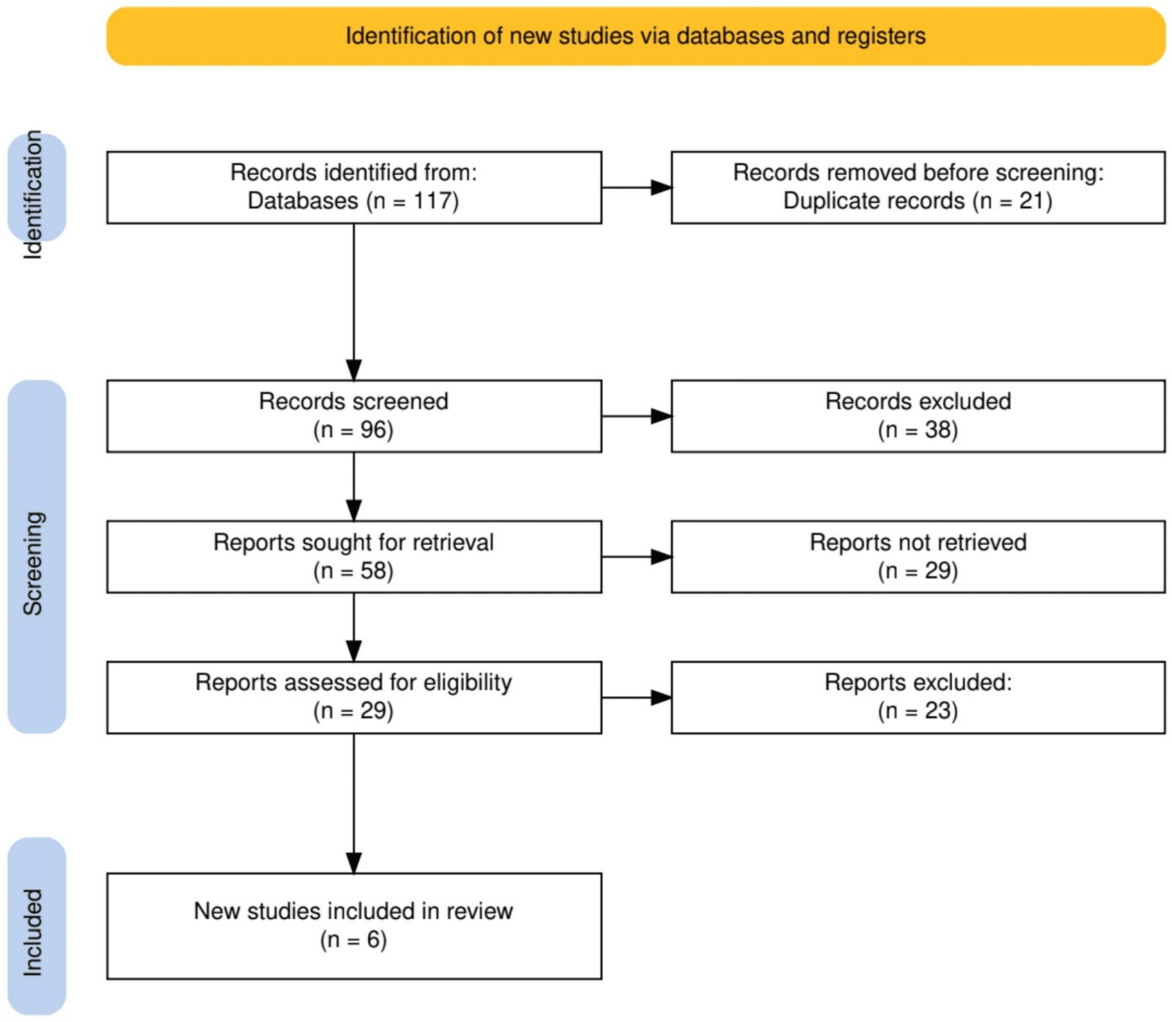
The PRISMA flowchart represents the process of study selection. PRISMA: Preferred Reporting Items for Systematic Reviews and Meta-Analyses.

The study selection process for our systematic review involved a meticulous and structured approach to ensure the inclusion of relevant and high-quality studies. Initially, a total of 117 records were identified from various databases, from which 21 duplicate records were removed before screening. The remaining 96 records were screened based on their titles and abstracts, leading to the exclusion of 38 records that did not meet the preliminary criteria. Of the 58 records deemed potentially relevant, full texts were sought; however, 29 reports could not be retrieved. The remaining 29 reports underwent a detailed eligibility assessment, resulting in the exclusion of 23 reports due to failure to meet specific inclusion criteria or due to quality concerns. Ultimately, six new studies met all eligibility criteria and were included in the review, providing a robust foundation for analyzing the effectiveness of neuroprotective strategies in enhancing post-stroke recovery. This process highlights the rigorous methodological standards adhered to in selecting studies for the review, ensuring the reliability and validity of the synthesized outcomes. A summary of the study selection process is given in Figure 1.

Characteristics of the Selected Studies

Table 1 provides a detailed overview of the key studies included in this systematic review, summarizing essential information such as the study purpose, methods, sample size, interventions, primary outcomes, key findings, and the associated risk of bias. The table is designed to offer a quick comparison of the effectiveness of neuroprotective interventions like SSRIs, Cerebrolysin, normobaric oxygen, and lithium in stroke recovery. Each study is listed with specific details, including statistical significance and clinical relevance, allowing for an easier evaluation of the variability in study designs and outcomes across different trials. This table complements the narrative synthesis by providing concise yet critical data points, aiding in the interpretation of the review’s overall findings.

**Table 1:** The table represents the key studies included in the systematic review. AIS: Acute Ischemic Stroke; ARAT: Action Research Arm Test; BI: Barthel Index; CARS: Cerebrolysin and Recovery from Stroke; CENTRAL: Cochrane Central Register of Controlled Trials; EMBASE: Excerpta Medica Database; FLAME: Fluoxetine for Motor Recovery After Acute Ischemic Stroke; MEDLINE: Medical Literature Analysis and Retrieval System Online; mRS: Modified Rankin Scale; MW: Mann-Whitney; NBO: Normobaric Oxygen; NIH: National Institutes of Health; NIHSS: National Institutes of Health Stroke Scale; NNT: Number Needed to Treat; PRISMA: Preferred Reporting Items for Systematic Reviews and Meta-Analyses; PsycINFO: Psychological Information Database; RCTs: Randomized Controlled Trials; SSRIs: Selective Serotonin Reuptake Inhibitors.

Article Citation	Study Purpose	Methods	Participants	Intervention	Primary Outcomes	Key Findings	Risk of Bias	Conclusions
Bornstein NM et al., 2018 [7]	To assess the efficacy of Cerebrolysin on global neurological improvement during the early post-stroke period.	Meta-analysis of nine randomized, double-blind, placebo-controlled trials. Data were synthesized using individual patient and aggregate data with the pre-planned and predefined Wei-Lachin robust pooling procedure.	1,879 patients across nine clinical trials.	30-50 ml Cerebrolysin administered daily for 10-21 days, starting within 72 hours of ischemic stroke onset.	Neurological status as measured by NIHSS at day 30 or 21, and mRS at day 90.	Significant improvement in NIHSS scores with Cerebrolysin compared to placebo (MW 0.60, P < 0.0001). - Clinically relevant changes in early NIHSS indicated a number needed to treat of 7.7. - Statistically significant favorable outcomes in mRS at day 90 in moderate to severe stroke patients (MW 0.61, P = 0.0118).	Not specified, but rigorous meta-analytic procedures suggest a controlled approach.	Cerebrolysin effectively improves early global neurological deficits in acute ischemic stroke patients, with safety comparable to placebo.
Mahmood A et al., 2022 [9]	To assess the effectiveness of normobaric oxygen (NBO) as a neuroprotective therapy in acute stroke.	Systematic review and meta-analysis of RCTs using MEDLINE, EMBASE, and CENTRAL up to December 2020.	9,255 participants across 12 cohorts.	Normobaric oxygen therapy administered <7 days after stroke to normoxic patients.	Early neurological recovery, functional outcomes, mortality, oxygen saturation, imaging markers.	No significant effect of NBO on reducing NIH Stroke Scale at 7 days. - No significant effect on modified Rankin Scale at 3-6 months or on mortality.	One major study with low risk of bias; smaller trials had limitations.	NBO showed no significant effect on early neurological recovery, functional outcome, or mortality. Studies predominantly used low-flow oxygen and commenced in the sub-acute phase.
Almeida OP et al., 2022 [10]	To review the evidence of lithium's neuroprotective and regenerative effects in animal models and human studies related to stroke.	Systematic review and meta-analysis following PRISMA guidelines, searching Medline, Embase, and PsycINFO from January 2000 to September 2021.	42 studies included: 36 rodent models of stroke and 6 human studies (4 meta-analyzed).	Lithium treatment in preinsult or postinsult phase.	Stroke volume, apoptosis, poststroke function in rodents; risk of stroke and clinical benefits in humans.	Human data showed a lower risk of stroke in bipolar disorder patients in 2 of 4 studies and equivocal benefits in clinical trials.	Not specified but assumed varied due to study designs and sample sizes.	Lithium shows promising results in animal models but lacks conclusive evidence in human studies, with a potential but untested role in human poststroke recovery.
Zhang D et al., 2017 [11]	To evaluate the efficacy and safety of Cerebrolysin in the treatment of acute ischemic stroke (AIS).	Meta-analysis of randomized controlled trials using PubMed, EMBASE, and the Cochrane Library. Interventions applied within 72 hours of stroke onset.	1,779 patients from 7 studies.	Cerebrolysin administration within 72 hours post-stroke.	Efficacy (assessed by mRS and BI scores) and safety (mortality and serious adverse events).	No significant efficacy of Cerebrolysin in improving mRS and BI scores. - Safety profile was neutral compared to placebo, with no significant differences in mortality or serious adverse events.	Potential publication bias and small number of studies may affect the accuracy of results.	Cerebrolysin appears safe but does not support its routine use for improving long-term rehabilitation after AIS based on current evidence.
Siepmann T et al., 2015 [12]	To explore the role of selective serotonin reuptake inhibitors (SSRIs) in improving clinical outcomes in patients following acute ischemic stroke, independent of their antidepressant effects.	Literature review using PubMed to identify evidence and mechanisms whereby SSRIs could enhance recovery from acute ischemic stroke.	Various clinical studies and a meta-analysis involving a total of 4,060 participants.	Administration of SSRIs, specifically focusing on fluoxetine in the context of the FLAME study.	Recovery of motor function and general stroke recovery.	FLAME study indicated significant motor recovery in stroke patients receiving fluoxetine. - Meta-analysis supported positive effects of SSRis on stroke recovery.	Limited sample sizes, heterogeneous designs, and a lack of large-scale randomized controlled trials comparing different SSRIs.	SSRIs, especially fluoxetine, show promise in aiding recovery from acute ischemic stroke. However, more comprehensive clinical trials and further elucidation of mechanisms are needed to translate these findings into clinical practice.
Guekht A et al., 2017 [13]	To assess the efficacy of Cerebrolysin in motor recovery during early rehabilitation post-stroke.	Meta-analysis of two identical, prospective, randomized, double-blind, placebo-controlled trials (CARS-1 and CARS-2). Treatment involved 30 ml Cerebrolysin daily for 3 weeks starting within 24-72 hours of stroke onset, alongside a standardized rehabilitation program.	442 patients from two clinical trials.	Cerebrolysin administered for 3 weeks post-stroke onset, combined with standardized rehabilitation.	Motor function recovery measured by ARAT score at day 90 and early neurological improvement by NIHSS at days 14 and 21.	Significant improvement in ARAT score at day 90 with Cerebrolysin compared to placebo (MW 0.62, P < 0.0001). - Early improvement by NIHSS at days 14 and 21 also showed statistical significance (MW 0.59, P < 0.002). - The number-needed-to-treat (NNT) for early NIHSS improvements was 7.1.	Controlled designs suggest a low risk of bias; however, the pooling method and conditions pre-defined under blinded conditions indicate rigorous methodological control.	Cerebrolysin significantly enhances motor function and neurological status in the early rehabilitation phase after an acute ischemic stroke, with safety comparable to placebo and a favorable benefit/risk ratio.

Discussion

The systematic review synthesized data from six studies evaluating the efficacy of various neuroprotective strategies for improving recovery in patients following acute ischemic stroke. These interventions included normobaric oxygen, lithium, Cerebrolysin, and SSRIs, particularly fluoxetine. Key findings across these studies revealed a spectrum of efficacy: normobaric oxygen therapy did not significantly affect early neurological recovery, functional outcomes, or mortality; lithium showed promising results in rodent models but lacked conclusive evidence in human studies; and SSRIs, specifically fluoxetine, demonstrated significant improvements in motor recovery. Cerebrolysin, examined in multiple studies, consistently showed improvement in early global neurological deficits and motor function during the early post-stroke period, with a notable number needed to treat of 7.1 for early improvements in neurological health as measured by the NIH Stroke Scale [7].

Collectively, these findings illustrate the varied potential of different neuroprotective agents in enhancing stroke recovery, highlighting Cerebrolysin’s particularly strong evidence for efficacy [14]. However, the overall effectiveness of these therapies in clinical settings remains mixed, with some showing more promise than others. The disparate results underscore the complexity of stroke recovery mechanisms and the need for tailored therapeutic approaches based on individual patient profiles and specific stroke characteristics [15]. This review also reveals the critical need for more large-scale, well-controlled clinical trials to further validate and refine the use of these neuroprotective strategies in routine clinical practice.

The contextualization of our review's findings within the existing literature reveals both concordance and divergence in the effectiveness of neuroprotective strategies for post-stroke recovery. The studies, like those by Mahmood A et al. and Almeida OP et al., have demonstrated a lack of significant improvement with normobaric oxygen and inconclusive effects of lithium that question the efficacy of these treatments in clinical settings [9,10]. In contrast, research supporting the use of Cerebrolysin and SSRIs, particularly fluoxetine, has often reported benefits in enhancing neurological recovery, corroborating our observations of their potential utility [16,17]. Such similarities may stem from consistent methodologies across studies, such as the use of randomized controlled trial designs and similar outcome measures like the NIH Stroke Scale [18] and modified Rankin Scale [19]. However, differences in findings could be attributed to variations in the timing of intervention administration, the dosage of treatments, and the demographic and geographical diversity of study populations, which can influence treatment efficacy and patient outcomes.

Moreover, the literature suggests a growing recognition of the need for a personalized approach to stroke therapy, acknowledging that the heterogeneity of stroke pathology and patient-specific factors can markedly affect treatment outcomes. For example, studies involving SSRIs have shown variability in efficacy based on the timing of administration post-stroke and the baseline severity of patients' conditions [20]. This is in line with our analysis, which highlights how patient selection and study demographics can impact the generalizability and effectiveness of neuroprotective agents. Additionally, the lack of large-scale studies and the variation in risk of bias across different research contribute to the discrepancies observed. As such, while our review adds to the growing body of evidence in support of certain interventions, it also emphasizes the necessity for more nuanced and robustly designed future studies to better delineate the circumstances under which these therapies are most beneficial.

The effectiveness of SSRIs and Cerebrolysin in stroke recovery demonstrated mixed but noteworthy results in the studies we analyzed. Fluoxetine, in particular, was associated with significant motor recovery, as evidenced in the FLAME trial and confirmed by a meta-analysis, with SSRIs showing moderate efficacy in improving neurological outcomes (P < 0.05) [12]. However, larger-scale randomized controlled trials are needed to validate these findings, especially for other SSRIs. On the other hand, Cerebrolysin consistently showed statistically significant improvements in early global neurological deficits and motor function, particularly in moderate to severe stroke patients, with a Mann-Whitney effect size of 0.60 (P < 0.0001) for the NIH Stroke Scale and a clinically meaningful number-needed-to-treat (NNT) of 7.1 [7,13]. While Cerebrolysin's efficacy was most pronounced in the early post-stroke phase, long-term rehabilitation benefits were less conclusive, with some studies indicating neutral outcomes for mRS and BI scores [11]. These findings suggest that while both SSRIs and Cerebrolysin offer potential benefits in acute and early rehabilitation phases, their long-term effects remain variable, warranting further large-scale, long-term trials to establish clear treatment guidelines.

The interpretation of our findings suggests that the varied efficacy of neuroprotective strategies like normobaric oxygen, lithium, SSRIs, and Cerebrolysin can be attributed to their differing mechanisms of action and their interaction with the complex pathophysiology of ischemic stroke. Normobaric oxygen, which failed to show significant improvement in our review, is thought to potentially increase oxygen availability to ischemic tissues, yet this might not sufficiently mitigate the cascade of cellular events leading to neuronal death [21]. In contrast, Cerebrolysin, showing more consistently positive outcomes, contains peptides that are hypothesized to directly enhance neurotrophic activity, potentially aiding neurogenesis and reducing apoptosis in the ischemic penumbra [22]. SSRIs like fluoxetine might promote recovery by modulating the inflammatory response and enhancing neuroplasticity, which aligns with their observed benefits in motor function recovery [6]. The contrasting results for lithium, showing promise in preclinical models but less so in clinical trials, might be due to differences in biological responses between species or possibly the insufficiency of translating preclinical dosing and timing to human subjects [23]. This variation in outcomes across different interventions underscores the complexity of stroke recovery, where multiple interlinked pathological processes may require distinct therapeutic approaches, tailored not just to the type of stroke but also to individual patient characteristics such as genetic predispositions and existing comorbidities.

The findings of this systematic review hold significant implications for both clinical practice and future research in the management of stroke recovery. Practically, the demonstrated efficacy of Cerebrolysin and SSRIs in improving post-stroke outcomes suggests that these treatments could be incorporated into existing stroke rehabilitation protocols, potentially enhancing patient recovery rates and quality of life [24]. Theoretically, the mixed results observed across different neuroprotective strategies underscore the complexity of stroke pathophysiology and the need for personalized treatment approaches, highlighting the importance of considering individual patient characteristics and stroke specifics when designing therapeutic regimens. For policy-making, these findings advocate for the support of further large-scale, multi-center clinical trials to robustly assess the utility and cost-effectiveness of these therapies in diverse populations. Additionally, the insights gained could guide the development of more nuanced guidelines that tailor neuroprotective interventions based on timely and precise diagnostic criteria. Ultimately, by advancing our understanding of how different neuroprotective agents function and their applicability in clinical settings, this research paves the way for more effective and targeted therapeutic strategies in stroke care, potentially reducing the global burden of this devastating condition.

The systematic review is strengthened by its comprehensive synthesis of data from multiple rigorously conducted studies, offering a broad perspective on the effectiveness of various neuroprotective strategies in stroke recovery. This breadth allows for a robust comparison and contrast of outcomes across different interventions and study populations, enhancing the reliability of our conclusions. However, the review also faces several limitations that impact the generalizability of the findings. One significant limitation is the variability in methodological quality among included studies, which ranges from high-quality randomized controlled trials to smaller, less rigorously controlled studies. This variability could introduce bias and affect the consistency of the results. Additionally, the heterogeneity in patient populations, stroke severity, and timing of intervention across studies complicates the direct comparison of results and may limit the applicability of findings to all stroke patients. Furthermore, most included studies were conducted in high-resource settings, which might not accurately reflect outcomes in lower-resource environments, thus limiting the global applicability of the findings. Acknowledging these limitations is crucial for interpreting the results with an appropriate level of caution and for guiding future research to address these gaps.

Future research should focus on addressing specific gaps in the understanding of neuroprotective strategies in stroke recovery. Key research questions include: (1) What are the optimal dosing regimens and timing for interventions such as SSRIs and Cerebrolysin to maximize their efficacy in both the acute and subacute phases of stroke recovery? (2) How do patient-specific factors, such as comorbidities or genetic profiles, influence the response to these neuroprotective therapies? (3) Can SSRIs and Cerebrolysin provide long-term benefits beyond motor recovery, such as improving cognitive and functional outcomes? Additionally, practical considerations for implementing these therapies in clinical settings include ensuring standardized treatment protocols, addressing potential side effects, and integrating these treatments within existing stroke rehabilitation frameworks [25,26]. Clinicians must also consider cost-effectiveness and the availability of these therapies, particularly in low-resource settings. Rigorous, large-scale, multicenter trials are essential to develop clear clinical guidelines and refine patient selection criteria, ensuring that neuroprotective therapies can be safely and effectively implemented in diverse patient populations.

## Conclusions

This systematic review critically evaluates the efficacy of various neuroprotective strategies in enhancing recovery following acute ischemic stroke, with a particular focus on Cerebrolysin, SSRIs, lithium, and normobaric oxygen. Our findings reveal the variable effectiveness of these interventions, highlighting the complexity of stroke recovery and emphasizing the need for personalized therapeutic approaches. Given the demonstrated benefits of Cerebrolysin and SSRIs in motor recovery and early neurological improvement, we recommend considering these therapies in the acute rehabilitation phase, particularly for moderate to severe stroke patients. Clinicians should tailor treatment plans based on individual patient profiles, including stroke severity, comorbidities, and timing of intervention, to maximize efficacy. Practical integration into clinical practice should involve standardized dosing protocols, close monitoring for side effects, and consideration of cost-effectiveness, particularly in resource-limited settings. However, further large-scale, high-quality studies are crucial to address existing knowledge gaps and ensure the safe, effective, and globally applicable implementation of these neuroprotective strategies. By addressing these challenges, future research can contribute to improved outcomes for stroke survivors worldwide.
